# Online-adjusted evolutionary biclustering algorithm to identify significant modules in gene expression data

**DOI:** 10.1093/bib/bbae681

**Published:** 2025-01-02

**Authors:** Raúl Galindo-Hernández, Katya Rodríguez-Vázquez, Edgardo Galán-Vásquez, Carlos Ignacio Hernández Castellanos

**Affiliations:** Instituto de Investigaciones en Matemáticas Aplicadas y en Sistemas, Universidad Nacional Autónoma de México, Circuito Escolar, Ciudad Universitaria, 04510 Mexico city, México; Instituto de Investigaciones en Matemáticas Aplicadas y en Sistemas, Universidad Nacional Autónoma de México, Circuito Escolar, Ciudad Universitaria, 04510 Mexico city, México; Instituto de Investigaciones en Matemáticas Aplicadas y en Sistemas, Universidad Nacional Autónoma de México, Circuito Escolar, Ciudad Universitaria, 04510 Mexico city, México; Instituto de Investigaciones en Matemáticas Aplicadas y en Sistemas, Universidad Nacional Autónoma de México, Circuito Escolar, Ciudad Universitaria, 04510 Mexico city, México

**Keywords:** biclustering, evolutionary algorithm, gene expression data, RNA-sequencing, single-cell RNA-sequencing

## Abstract

Analyzing gene expression data helps the identification of significant biological relationships in genes. With a growing number of open biological datasets available, it is paramount to use reliable and innovative methods to perform in-depth analyses of biological data and ensure that informed decisions are made based on accurate information. Evolutionary algorithms have been successful in the analysis of biological datasets. However, there is still room for improvement, and further analysis should be conducted. In this work, we propose Online-Adjusted EVOlutionary Biclustering algorithm (OAEVOB), a novel evolutionary-based biclustering algorithm that efficiently handles vast gene expression data. OAEVOB incorporates an online-adjustment feature that efficiently identifies significant groups by updating the mutation probability and crossover parameters. We utilize measurements such as Pearson correlation, distance correlation, biweight midcorrelation, and mutual information to assess the similarity of genes in the biclusters. Algorithms in the specialized literature do not address generalization to diverse gene expression sources. Therefore, to evaluate OAEVOB’s performance, we analyzed six gene expression datasets obtained from diverse sequencing data sources, specifically Deoxyribonucleic Acid microarray, Ribonucleic Acid (RNA) sequencing, and single-cell RNA sequencing, which are subject to a thorough examination. OAEVOB identified significant broad gene expression biclusters with correlations greater than $0.5$ across all similarity measurements employed. Additionally, when biclusters are evaluated by functional enrichment analysis, they exhibit biological functions, suggesting that OAEVOB effectively identifies biclusters with specific cancer and tissue-related genes in the analyzed datasets. We compared the OAEVOB’s performance with state-of-the-art methods and outperformed them showing robustness to noise, overlapping, sequencing data sources, and gene coverage.

## Introduction

Analyzing a large amount of gene expression data can be challenging for conventional mathematical methodologies. However, computer algorithms allow the modeling of gene coordination in response to changing environmental conditions, providing valuable insights into gene expression [1]. This knowledge has driven significant advancements in medicine and biotechnology.

Gene expression involves transcription and translation. During transcription, Deoxyribonucleic Acid (DNA) is transferred to Ribonucleic Acid (RNA), while translation involves decoding RNA into proteins [2–8].

There are different ways to measure gene expression, including methods operating on a smaller scale, such as Serial Analysis of Genetic Expression or Polymerase Chain Reaction [9, 10]. However, DNA Microarray and RNA-sequencing (RNA-seq) are two high-throughput methods that have come into usefulness [11]. Additionally, single-cell RNA-sequencing technologies allow for unbiased, high-throughput, and high-resolution transcriptome investigations of individual cells. Single-cell RNA sequencing has provided insights into tissue composition, transcription dynamics, and gene regulatory networks. Several gene expression databases cover bacteria, plants, and humans, including repositories relevant to diseases like cancer [12–17]. The Sequence Read Archive, for instance, contains high-throughput data that has grown exponentially to approximately $36$ petabytes (https://ncbiinsights.ncbi.nlm.nih.gov/2020/06/30/sra-rfi/).

The abundance of these gene expression datasets allows for finding condition-specific functional gene modules, defined as a highly organized expression pattern on a certain gene set. These modules are often associated with particular biological processes, such as diseases. In this context, biclustering techniques have been increasingly used to analyze gene expression data [18–20]. Evolutionary algorithms have proven particularly helpful in this context, as they help identify meaningful relationships and group common data in both rows and columns [21]. Biclustering aims to uncover relationships in the data and cluster specific rows and columns in any order. However, solving the biclustering problem and finding significant biclusters is difficult as it falls into the NP-hard category [22].

This work proposes a novel Online-Adjusted EVOlutionary Biclustering algorithm (OAEVOB) to analyze gene expression data and identify significant gene groupings involved in biological functions. OAEVOB is capable of identifying crucial biological relationships in any numerical gene expression matrix. The main contributions of our work are the following:

- The method’s primary innovation is the application of the Jaccard coefficient to eliminate nearly identical biclusters and using two quality measu-rements, $ACV$ (Average Correlation Value) and $VE^{t}$ (Transposed Virtual Error) in the fitness evaluation and mutation process, respectively [23]. The $ACV$ calculation effectively detects enriched biclusters and has a relationship with gene ontology (GO) terms.
- We conducted experiments to determine the most suitable parameters for improving OAEVOB’s performance, including preprocessing types, online-adjustment techniques for crossover and mutation, similarity measurements, initial exploration, and mutation types.
- OAEVOB includes most of the genes in the biclusters from the analyzed data, allowing gene analysis to identify meaningful biological relationships.
- Using a gene enrichment analysis, we assessed the biclusters obtained by OAEVOB and discovered significant modules related to cancer types and specific tissues.
- OAEVOB can identify significant modules in any numerical gene expression matrix, regardless of the technology used to extract the expression information.
- OAEVOB obtained competitive results in relevance and recovery scores compared to the state-of-the-art algorithms in simulated datasets (SD).
- We compared OAEVOB with state-of-the-art algorithms and achieved competitive results in identifying significant biclusters and relevant biological functions.

The document is structured as follows. The second section describes the state-of-the-art algorithms in the specialized literature. The third part explains the gene expression datasets used in this work and outlines the OAEVOB’s stages. The following section shows the results and compares them with state-of-the-art biclustering algorithms. The fifth section describes the gene enrichment analysis of the modules obtained by OAEVOB. The sixth section analyzes the results and the last section discusses the conclusions and future work.

## State-of-the-art

Many metaheuristic approaches have been developed to identify significant gene patterns, and in this section, we will highlight the most relevant papers focusing on biclustering algorithms.

In [24], ELSA was proposed to evaluate biclusters’ statistical and biological quality separately, using two objective functions based on the Average Correlation Function (ACF). An archiving strategy and two different types of mutations are used in ELSA. The first mutation type selects only annotated genes, which may result in the loss of significant genes by ignoring certain genes. The second mutation type involves incorporating unannotated genes into biclusters. Compared to other algorithms, ELSA performs better on microarray datasets. However, ELSA may force the formation of biclusters with false relationships. Additionally, gene information should be excluded to avoid bias towards known genes and uncover interesting relationships that have not yet been discovered.

In [25], the authors introduced EBA. The researchers used biclustering quality indicators: bicluster size (BSize), Mean Square Residue (MSR), and ACF. It was found that the EBA configuration that uses selection with aggregation and biclustering crossover and mutation operators outperformed other configurations for microarray datasets. In addition, EBA and ELSA evaluated the algorithms’ performance using the same two microarray datasets without using RNA-seq and single-cell RNA-seq data, which is significant since these are nowadays the dominant techniques for gene expression analysis.

In [26], Bi-Phase Evolutionary Searching for Biclusters (BP-EBA) employed binary encoding, hierarchical clustering to find bicluster seeds, and biclustering quality indicators such as MSR, Scaling MSR, and BSize. The algorithm operates in two stages to evolve genes and conditions. BP-EBA was compared to previous biclustering algorithms using microarray datasets, and the overall results were good. However, during the process of forming bicluster seeds, crucial genes and conditions might be excluded, impeding meaningful data analysis.

In [27], the researchers introduce QUBIC2, which generates a representing gene for each row in the discretization matrix. Bicluster seeds are detected and expanded by identifying genes that improve the bicluster. The study shows that QUBIC2 outperforms other algorithms like EBIC, BIMAX, ISA, and PLAID on microarray, RNA-seq, and single-cell RNA-seq datasets [18]. However, a mixture of Gaussian distribution (MGD) is recommended for microarray datasets, while left-truncated MGD is recommended for RNA-seq-based datasets. Hence, two separate algorithms were developed to address the biclustering task for different sequencing data.

In [28], RecBic generates bicluster seeds by considering each subset of columns. The highest trend-preserving biclusters with dimensions of $h*3$ are identified based on each pair of columns in each subset. It then extends the core bicluster with a preset error rate $\alpha $ when there is noise while following the trend-preserving approach to extend the bicluster without noise. RecBic outperformed QUBIC2 in finding significant biclusters using microarray and RNA-seq datasets. However, RecBic’s complexity is $O(n^{3})$ in the main configuration, which might be highly complex when using large datasets.

All-round biclustering algorithm (ARBic) [29] is presented as an all-around biclustering algorithm to handle noise levels and trend preservation in datasets. The authors use a pseudo directed acyclic graph to detect the longest path in the seeds and grow the optimum seeds to form biclusters. The authors analyze five real datasets (yeast, *Escherichia coli*, and human) with many columns to identify biclusters that are broader and not narrow (biclusters with a few columns), incorporating several columns in biclusters rather than being limited to just a few. ARBic found trend-preserved and overlapped biclusters with different noise levels in SDs, outperforming QUBIC2. However, ARBic is primarily applied to microarray datasets, lacking analysis on RNA-seq and single-cell RNA-seq real datasets. These datasets typically contain many columns and should have been utilized for their comparison to identify broader biclusters. Furthermore, ARBic utilizes RecBic for datasets with fewer than $500$ columns and greater than $10\,000$ rows, demonstrating that RecBic outperforms ARBic in these datasets.

RUBic [30] is a biclustering algorithm that prioritizes speed and scalability. RUBic’s input is a binary matrix that is transformed to decimal by converting consecutive four-bit binary numbers encoded from a pair of seed rows using bit-wise AND operations. However, RUBic is designed to extract biclusters from binary datasets, lacking analysis of a large number of microarray, RNA-seq, and single cell RNA-seq datasets that are not binary. BGB [31] utilizes biological graph knowledge, providing control over the correlation level of the shrinkage parameters and allowing for the retrieval of overlapping biclusters. Nevertheless, it is a biclustering algorithm designed to extract biclusters from binary datasets, similar to RUBic.

In [32], a two-stage biclustering algorithm using NSGA-II was employed to find sparse biclusters in microarray datasets. This algorithm addresses two objectives: bicluster size and $ACV$. They utilized NSGA-II was employed to find sparse biclusters in microarray datasets. The algorithm outperformed other biclustering algorithms in finding scale and shifting patterns in noisy synthetic and real datasets. However, our paper focuses on OAEVOB using a single objective. Future work will consider many objectives in the biclustering task.

We analyzed various biclustering algorithms and their variations and believe there is room for improvement, especially in using RNA-seq and single-cell RNA-seq datasets. We propose a novel algorithm to address these concerns. The OAEVOB’s components are further described in subsection (Online-Adjusted EVOlutionary Biclustering).

## Materials and methods

### Gene expression data

Gene expression data extraction technologies vary significantly due to differences in experimental technologies and the nature of the information captured. For instance, microarrays have been widely used since the late 90s, and although it is no longer very popular, a large amount of data are available [33]. RNA-seq technology is more accurate than microarrays in determining gene expression values [34]. Furthermore, researchers have found single-cell RNA-seq technology to be effective at identifying gene functions that were previously undetected using microarrays and RNA-seq [35].

Given the importance of these varied technologies, analyzing gene expression datasets using biclustering algorithms to detect patterns is crucial for uncovering novel relationships. To comprehensively assess biclustering algorithms, we carefully selected six diverse datasets: Tissp, Cocel, Mouse, Ustilago, BCancer, and GPL5175, described in Table 1, representing different sequencing technologies and species (human, *Ustilago maydis*, and mouse). These datasets offer a significant challenge in identifying tissue-specific genes, cancer-related genes, and brain functions.

**Table 1:** Datasets analyzed by OAEVOB

**Dataset**	**Technology**	Genes ^*^ Conditions	**Size**	**Description**
Tissp	DNA microarray	$14\,126*158$	$33.7$ MB	Gene expression profiles of $79$ physiologically healthy human tissues.
Cocel	RNA-seq	$14\,200*54$	$23.9$ MB	$54$ human cell lines of colon-rectal cancer.
Mouse	Single-cell RNA-seq	$3005*5000$	$30.6$ MB	S1 and CA1 region, from $3005$ single-cell transcriptomes.
Ustilago	DNA microarray	$5810*168$	$11.8$ MB	*Ustilago maydis* genes.
BCancer	RNA-seq	$28\,143*49$	$22.3$ MB	Breast cancer genes.
GPL5175	DNA microarray	$2308*4436$	$184.9$ MB	Human tissue genes.

The first dataset, Tissp, contains gene expression data from tissue-specific of seventy-nine pathologically healthy human tissues identified with microarray technologies. It includes $14\,042$ human genes and $158$ samples [14]. Also, we annotated each gene using an extensive Ensemble ID database for unique identification www.ncbi.nlm.nih.gov/sites/GDSbrowser?acc=GDS596.

The second dataset, the Colon-rectal Cancer dataset (here called Cocel), comprises $54$ cell lines and $14\,200$ genes from RNA-sequencing samples of various malignancies collected from the Cancer Cell Line Encyclopedia [36]. Thus, colon-rectal cancer receives special attention in our work; the necessary cell lines were screened www.ncbi.nlm.nih.gov/geo/query/acc.cgi?acc=GSE35896.

In the third dataset, Mouse, scientists used single-cell RNA-sequencing to analyze mice’s primary somatosensory cortex and hippocampus $CA1$ region [37]. Mouse contains $3005$ genes and $5000$ samples https://www.ncbi.nlm.nih.gov/geo/query/acc.cgi?acc=GSE46980.

GPL5175 is a human tissue collection aggregating public datasets utilizing the GPL5175 microarray platform https://zenodo.org/records/1157938. It was obtained from seek.princeton.edu and used in [29]. Ustilago is a microarray dataset of *U. maydis* including $5810$ genes and $168$ conditions [38]. Breast cancer is a RNA-seq dataset that contains $28\,143$ genes and $49$ conditions (here called BCancer) [39].

Therefore, analyzing these datasets using biclustering algorithms to detect patterns is crucial for identifying relationships in future datasets that can provide novel and significant information. We meticulously selected relevant datasets to gather diverse sequencing data to assess biclustering algorithms comprehensively.

### Online-Adjusted EVOlutionary Biclustering

This section introduces the fundamental components of OAEVOB. We conducted a broad analysis of the selected components and investigated the results for statistical significance.

#### Preprocessing

In preprocessing, data are cleaned and normalized. ELSA and QUBIC2 replace zero values with other values greater than zero. However, modifying the dataset might change the algorithm’s results. In our work, we explored alternatives to this treatment. We explored strategies individually to preprocess data: (1) Mean: replacing each value considering the row mean; (2) Standard deviation: replacing each value considering the standard deviation of the row; (3) Z-score: the values are normalized based on the mean and standard deviation; (4) Var-filter: R library to perform data curation with parameters to tune; (5) Remove zeros: it consists of removing rows that contain at least one zero; (6) Scalarization: Gaussian with zero mean and variance of one; (7) RPKM (Reads per Kilobase Million): it is employed in single-end RNA-seq, where every read corresponds to a sequenced single fragment; (8) FPKM (Fragments per Kilobase Million): the number of gene fragments is divided by the total sequencing depth; thus, the ratio is divided by the gene length. (9) TPM (Transcripts per Kilobase Million): normalization for gene length and sequencing depth to compare the proportion of reads mapped to a gene in each sample. Particular strategies outperform others in boosting OAEVOB outcomes. In summary, zero removal (ZR) outperformed the Mean, Var-filter, Standard Deviation, and Z-score in Tissp and Cocel. Only RNA-seq data can utilize RPKM, FPKM, and TPM. TPM outperformed RPKM and FPKM, likely due to its resemblance to scalarization. Therefore, ZR and scalarization were applied to Tissp, Cocel, Ustilago, BCancer, and GPL5175, and TPM was applied to Cocel and BCancer. Mouse does not undergo any preprocessing since single-cell RNA-seq datasets are typically highly dispersed and contain many zeros, rendering ZR ineffective.

#### Codification

**Figure 1:**
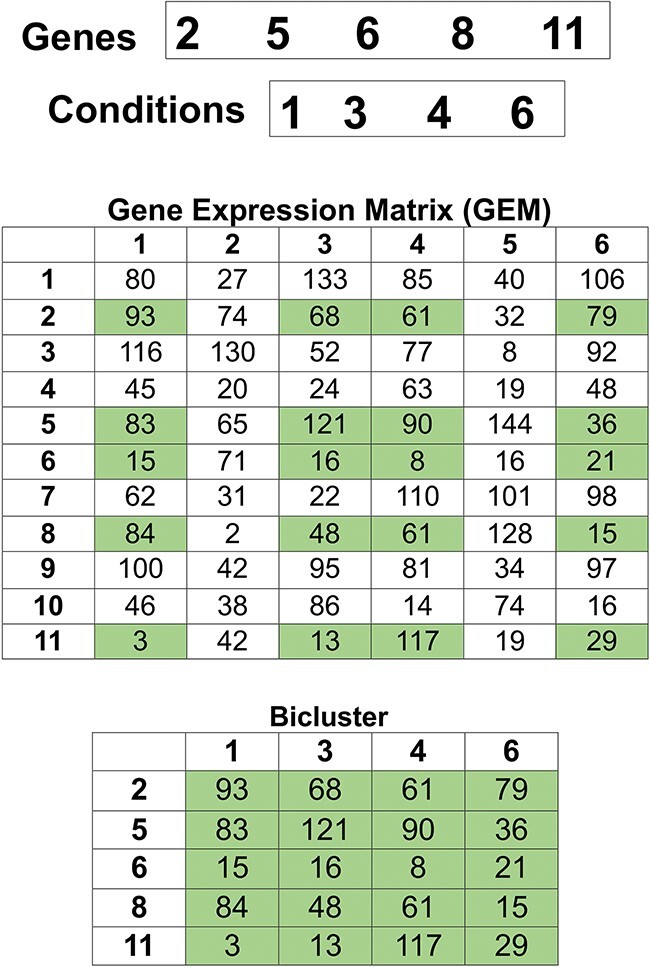
Representation of bicluster’s codification in OAEVOB. The indexes of genes ($2$, $5$, $6$, $8$, $11$) and conditions ($1$, $3$, $4$, $6$) are randomly selected to form the bicluster that contains the original values obtained from GEM (rectangles colored in green).

Differentiating between genes and conditions within a single string that encodes the bicluster and the presence of many zeros may make the gene expression analysis more difficult using high time and memory resources [20, 26, 40]. The encoding method used in this work comprises only the integer indices of genes and conditions within the bicluster. This approach avoids the limitations of binary strings, such as the need to have a length-fixed string with several zeros. This approach eliminates the need to differentiate between genes and conditions in a single string. Figure 1 illustrates how the genes and conditions are randomly selected to create a bicluster.

#### Initial exploration

In generation zero, OAEVOB creates biclusters using integer codification to explore hidden spanning patterns in the data. OAEVOB randomly proposes $300$ biclusters using MIXRNG (it combines the Python random number generator and a random number generator based on [41]). We determined to avoid creating many biclusters in the range of $600$–$3000$, as it was computationally expensive. After running OAEVOB $36$ times, it was found that creating large numbers of biclusters did not significantly improve the outcomes regarding the correlations we obtained (fitness). Statistical significance is enhanced by running OAEVOB $36$ times as suggested in [42] and [43]. Generally, $36$ runs is often considered a practical balance between computational cost and statistical robustness [43].

**Figure 2:**
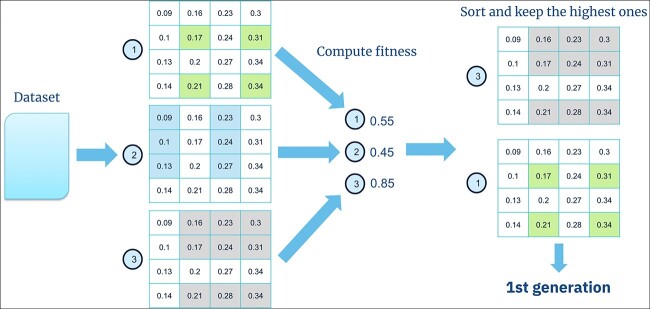
In this example, three biclusters are formed in the initial exploration. The fitness of each bicluster is then calculated, and the two biclusters with the highest fitness are preserved for the first generation.

The $300$ biclusters created during the initial exploration stage are sorted based on their fitness. Figure 2 shows the chosen genes and conditions in green, blue, and gray squares. We preserve $bic=120$ biclusters with the highest fitness to maintain a $population=60$ biclusters in the subsequent generations. The number of biclusters can not be determined in real datasets without ground truth. Since there is no fixed number of biclusters used in biclustering algorithms of the specialized literature, we use $60$ biclusters to establish a fixed number and have a fair comparison with the state-of-the-art algorithms.

The MIXRNG excels in generating different random numbers, thereby proposing significantly diverse biclusters. This diversity is crucial for exploring dissimilar biclusters within the dataset. With MIXRNG, the initial exploration process is straightforward, requiring only a single parameter to define the number of biclusters created, ensuring high-quality solutions. Contrarily, other methods necessitate the definition of multiple parameters, which can be challenging due to the various combinations in each dataset.

#### Crossover and mutation

We used Simulated Binary Crossover (SBX) for crossover [44], adapting it for use with integers by taking the floor of the number. Despite SBX performing well in codifications with numbers in $\mathbb{R}$, we employed it in $\mathbb{Z}$, as it can generate biclusters with high correlation.

Furthermore, OAEVOB uses three mutation types: addition, replacement, or removal of a gene or condition in a bicluster [18]. Thus, a random number is generated to decide which mutation to use in each iteration. A virtual condition ($VC_{t}$) and a virtual gene ($VG_{t}$) are computed in the mutation process. This calculation involves a detailed process of computing a representative gene and condition that best represents the bicluster. It is worth noting that this strategy can be achieved using the $VE^{t}$ measurement as described in [23]. The addition strategy adds a gene or condition that improves the bicluster’s fitness. The replacement strategy involves substituting a gene or condition in the bicluster that is underperforming based on its fitness; then, a gene or condition that improves the bicluster’s fitness is added. Similarly, the removal strategy deletes the gene or condition with the poorest fitness performance in the bicluster.

#### Adaptation of evolutionary operators

**Figure 3:**
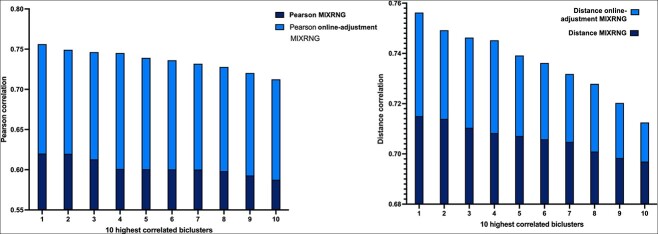
The highest fitness scores were obtained utilizing online-adjustment using Pearson and distance correlation.

**Figure 4:**
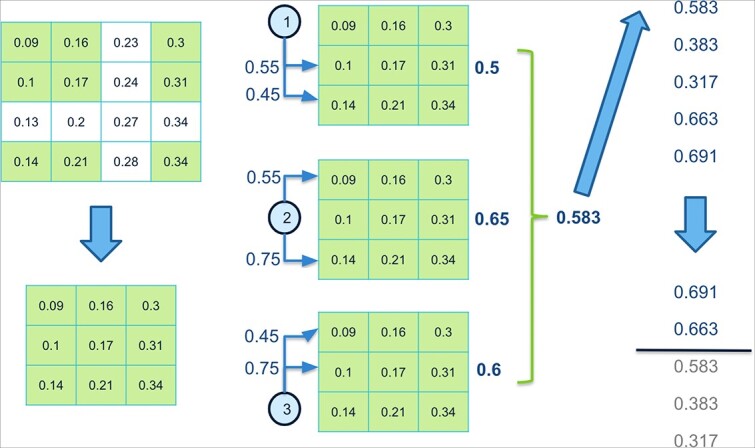
We compute the bicluster’s $ACV$. We calculate the correlation of each gene concerning the remaining genes in the bicluster and compute the average to determine the bicluster’s fitness ($0.583$). The biclusters are then sorted by fitness, and we choose the $bic=2$ (in this example) with the highest fitness.

**Figure 5:**
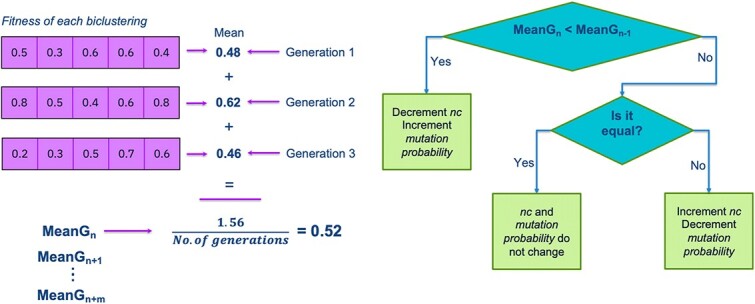
The $BicAv$ is the average of all the biclusters’ fitness of the last $k$ generations ($0.52$ in this example). When the current $BicAv$ is less or greater than the previous one, the online-adjustment characteristic updates the $\eta c$ and mutation probability values.

The bicluster’s fitness is determined by calculating a similarity measurement (Fig. 4). We sort the biclusters by fitness and consider top $bic=60$ for the fitness average $BicAv$ (Biclusters Average) computation (Fig. 5). The $BicAv$ is calculated every $k=5$ generations until a stop criterion is met. We tested other values for $k$ in a range from $k=2$ to $50$; however, these values worsened the OAEVOB’s results regarding correlations obtained. The online-adjustment characteristic involves updating the $\eta c$, which is a crucial value of the complete SBX operator, and mutation probability values regarding the $BicAv$ of the current generation. Variation in $\eta c$ influences the similarity of the offspring, resulting in them being very different or nearly identical, particularly in the range from $2$ to $30$, respectively, which is used in this work [44]. The online-adjustment also updates the mutation probability values, which are set to $1/30$ when OAEVOB individuals have greater fitness. These probabilities are increased to $0.1$ when the offspring exhibit poorer fitness in order to apply mutation to more individuals. Online-adjustment takes advantage of knowing the fitness of individuals to determine if OAEVOB is improving their biclusters and then to set a greater value to $\eta c$ (until $30$) and a value of $1/30$ to mutation probability to generate similar offspring. On the other hand, when the offspring have poorer fitness, the $\eta c$ value is decreased (until $2$), and mutation probability is increased to $0.1$ to generate very different individuals. Furthermore, online-adjustment was implemented in OAEVOB and compared when it was absent using Pearson and distance correlation. The results are illustrated in Fig. 3. The biclusters with the online-adjustment characteristic have the highest correlations in the top 10 biclusters, outperforming those without this characteristic.

The online-adjustment considers the $BicAv$ value to update $\eta c$ in the crossover process and the mutation probability. When the current $BicAv$ is less than the previous $BicAv$, the $\eta c$ value decreases, and the mutation probability increases for generating different offspring. When $BicAv$ is greater than the previous $BicAv$, the mutation probability decreases, and the $\eta c$ value increases, resulting in more similar offspring. These updates allow OAEVOB to continue exploring by proposing alternative biclusters and exploiting promising ones.

Algorithm 1 describes the updates of $\eta c$ and mutation probability values. The $\eta c$ value is crucial for the SBX operator. It ranges from 2 to 30 and changes the crossover process, creating different biclusters when the $\eta c$ value is low. Conversely, when the biclusters are of good quality, the $\eta c$ value is high, which helps to obtain biclusters that are similar to those of good quality.

**Table:** 

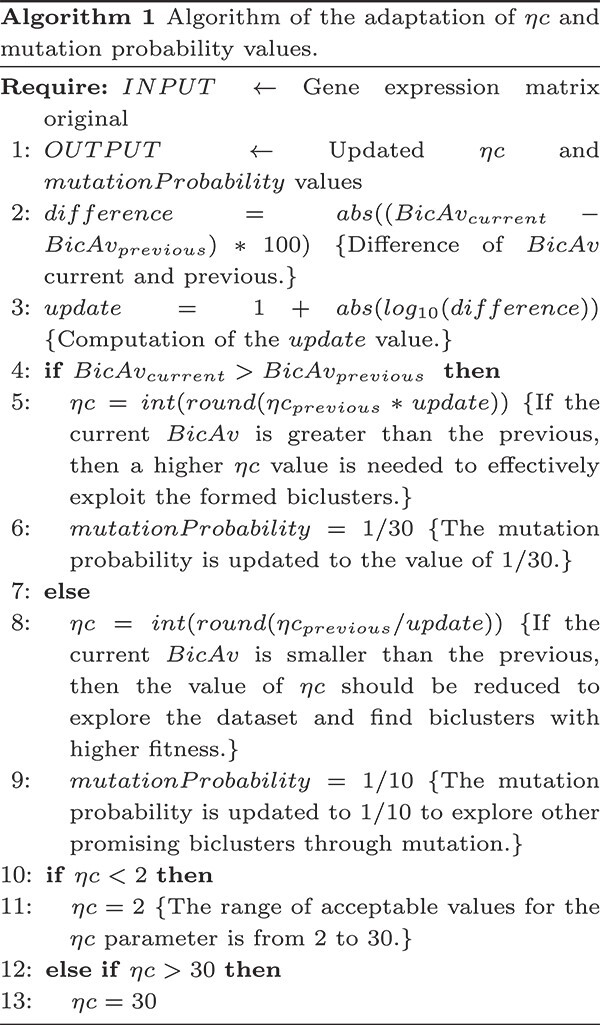

#### Fitness evaluation

The $ACV$ strongly correlates with GO terms, making it a useful tool for detecting enriched biclusters and identifying significant biological functions. OAEVOB uses the $ACV$ value to evaluate biclusters using four measurements: Pearson correlation, biweight midcorrelation, distance correlation, and mutual information. Each measurement contributes to our comprehensive assessment of the biclusters.

Pearson correlation measures the strength and direction of a linear relationship between two random variables [45]. However, it is susceptible to outliers. Our work considers Biweight midcorrelation to address this issue [46]. Another approach, distance correlation, was introduced in [47] to determine dependence and independence between two random vectors. Additionally, Mutual Information (MI) can be used to quantify how much knowledge one random variable has about another variable [48].

#### Jaccard coefficient computation

When performing generations, we obtain similar biclusters proposed by OAEVOB using MIXRNG. We used the Jaccard Coefficient (JC) computation to measure the similarity between biclusters and propose unique biclusters in each generation. OAEVOB compares each bicluster with all others in each generation to compute its JC by pair, resulting in a matrix of JCs with a diagonal of $1$. Our observation that biclusters with a JC greater than $0.3$ share several genes and conditions is a pivotal insight. We propose two thresholds: a soft threshold $=0.1$ (ST) and a hard threshold $=0.3$ (HT). If the JC between two biclusters is greater than ST, one is replaced with a new randomly created individual, and the other is kept ($replaceOneIndividual$ in Algorithm 2). If the JC is greater than HT, both individuals are replaced by two randomly created individuals ($replaceBothIndividuals$).

#### The complete OAEVOB algorithm

Algorithm 2 describes OAEVOB’s steps. Users specify upper and lower bounds for the number of rows and columns ($m*n$, lines $4$ and $5$), i.e. the number of genes and conditions that will form any bicluster, and the number of biclusters (line $2$). Our model begins (line $10$) by generating three hundred biclusters (individuals) using MIXRNG (line $11$). Fitness computation (line $12$) is computed employing a similarity measurement, and the top $120$ biclusters are preserved (line $13$) for the first generation (line $14$). This involves calculating the JC (lines $15-20$) and selecting individuals for crossover and mutation (lines $21-23$).

Subsequently, the $BicAv$ value of the current generation is calculated and compared against the previous one (lines $24--25$ and $28$). The $\eta c$ and $mutation$$probability$ values are adjusted (lines $26--27$). The biclusters are sorted concerning their fitness (lines $29--30$). The algorithm ends when a stop criterion is reached (the number of generations $=100$).

The OAEVOB’s algorithm complexity is $O(n^{2})$ when employing Pearson and biweight midcorrelation, where $N = GEM$ (n rows ^*^ m columns). It is $O(n^{3})$ when using MI and distance correlation.

**Table:** 

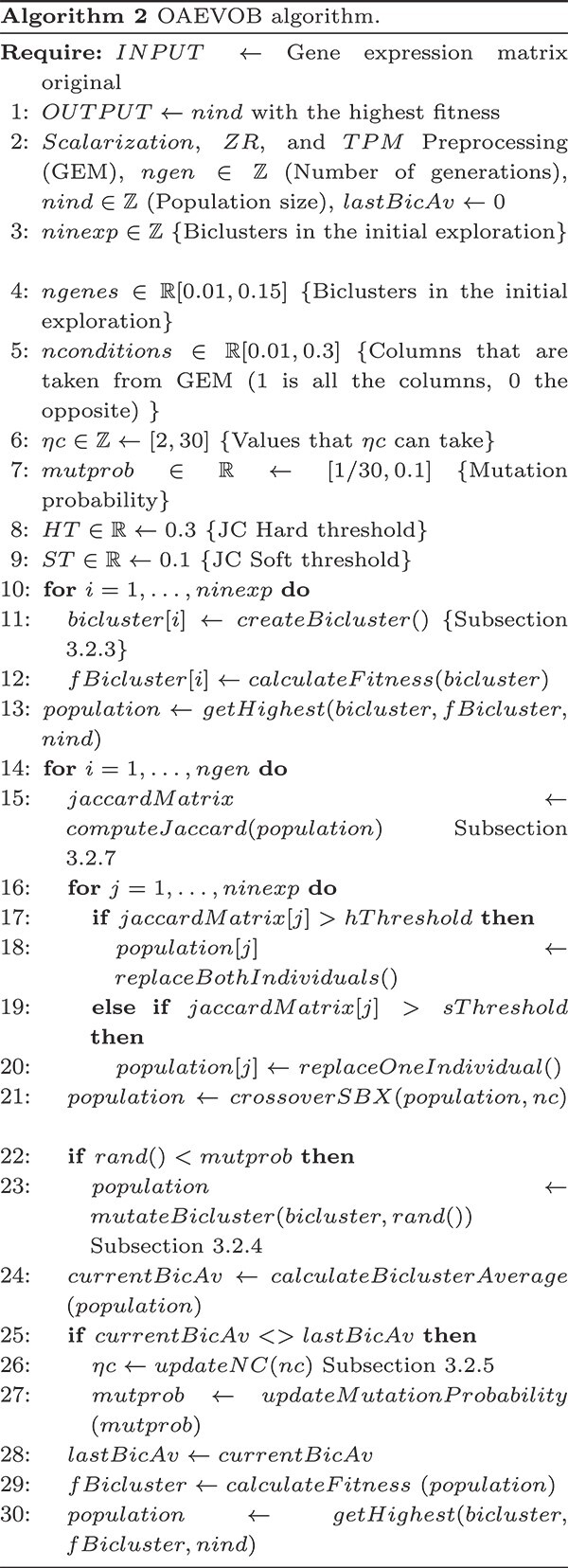

**Figure 6:**
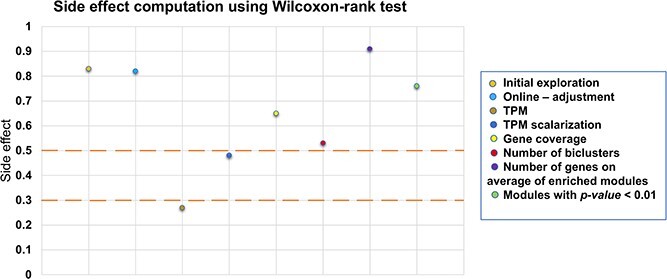
Wilcoxon-rank test to compute the side effect. In this case, a value greater than 0.3 is considered a medium level, and a greater than 0.5 is strong. The features of initial exploration, online-adjustment, and TPM scalarization (in Cocel) are shown to improve the OAEVOB’s performance.

**Figure 7:**
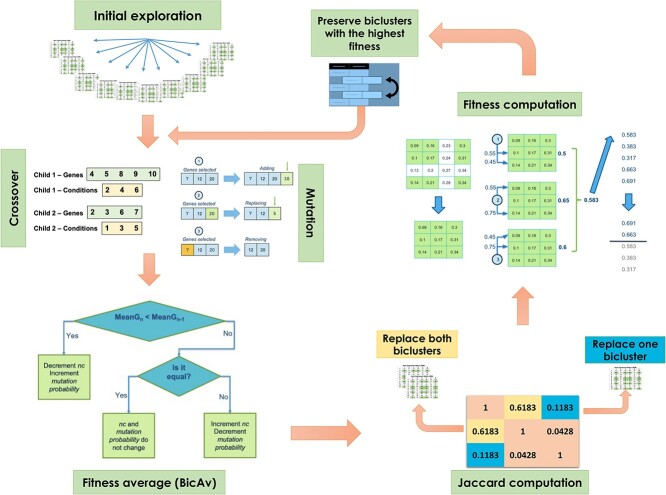
The main steps of OAEVOB for all the generations. The algorithm begins with the initial exploration performed only once. The following steps, which are performed in every generation, consist of crossover, mutation, Jaccard calculation, fitness, $ACV$ computation, and preserving the biclusters with the highest fitness.

To find the best parameter tuning, researchers have utilized statistical tests such as Wilcoxon-rank [49, 50]. Figure 6 shows the statistical significance of each parameter using Wilcoxon-rank. The outcomes indicated that MIXRNG yields better results during initial exploration, computing the Jaccard coefficient, preprocessing using scalarization, ZR and TPM, $\eta c$, and $mutation$$probability$ in their ranges, are the most suitable parameters for OAEVOB. OAEVOB is visually represented in Fig. 7.

## Results

This section presents the experimental settings and the results obtained by OAEVOB. For all experiments, we performed $36$ independent runs.

### Similarity measurements

The bicluster’s fitness is calculated using four similarity measurements. The highest fitness that OAEVOB reaches across all datasets is described in Table 2. MI values range from zero to infinite. Therefore, we randomly proposed thirty thousand unrepeated biclusters in the six datasets and computed their MI to normalize the values from zero to one. Only OAEVOB is analyzed in this subsection because its fitness is based on four similarity measurements. Table 3 describes the parameters used in the similarity measurements analysis.

**Table 2:** Highest fitness obtained by OAEVOB in the datasets

**Dataset**	**Pearson corr.**	**Biweight midcorr.**	**Distance corr.**	**Mutual Info.**
Tissp	0.872202 $\pm $ 0.03646	0.714466 $\pm $ 0.01358	0.807530 $\pm $ 0.01077	0.999639 $\pm $ 0.00065
Cocel	0.553548 $\pm $ 0.01403	0.526466 $\pm $ 0.00619	0.546173 $\pm $ 0.01786	0.999465 $\pm $ 0.00923
Mouse	0.548686 $\pm $ 0.02094	0.524901 $\pm $ 0.01005	0.529108 $\pm $ 0.01163	0.992874 $\pm $ 0.00299
Ustilago	0.926253 $\pm $ 0.00492	0.859471 $\pm $ 0.00847	0.824986 $\pm $ 0.00682	0.999816 $\pm $ 0.00074
BCancer	0.890483 $\pm $ 0.02903	0.769392 $\pm $ 0.02193	0.748021 $\pm $ 0.03920	0.994935 $\pm $ 0.01092
GPL5175	0.700671 $\pm $ 0.02799	0.623995 $\pm $ 0.01906	0.620129 $\pm $ 0.02363	0.991038 $\pm $ 0.00539

Fitness normalized: $1$ is best and $0$ is worst. Average and $\pm $ standard deviation.

**Table 3:** Parameters configuration for the similarity measurements computation. Values depict percentages of rows or columns

**Parameter**	**Genes (rows)**	**Conditions (columns)**
Tissp	[$0.01$, $0.04$]	[$0.04$, $0.1$]
Cocel	[$0.01$, $0.04$]	[$0.1$, $0.3$]
Mouse	[$0.05$, $0.15$]	[$0.001$, $0.003$]
Ustilago	[$0.025$, $0.1$]	[$0.04$, $0.1$]
BCancer	[$0.005$, $0.02$]	[$0.1$, $0.3$]
GPL5175	[$0.05$, $0.15$]	[$0.001$, $0.003$]

**Table 4:** General parameters configuration of OAEVOB

**Parameter**	**Value**
Generations	100
Population	60
Genes (rows)	[0.005, 0.15]^*^
Conditions (columns)	[0.001, 0.3]^*^
Crossover	1
$\eta c$	[2, 30]
Mutation probability	[1/30, 0.1]
Similarity measurements	Pearson, Biweight, Distance and MI
Hard, soft threshold	0.3, 0.1
Random number generator	MIXRNG

$^{\ast }$ Values depict percentages of rows or columns.

### Relevance and recovery scores

We utilize recovery and relevance scores [51], based on the similarity and match score (average similarity) between two biclusters defined as [29]: 


\begin{align*}score(G_{1}, G_{2}) = \begin{array}{l}\frac{1}{|G_{1}|} \sum _{bic_{1} \in G_{1}} \max _{bic_{2} \in G_{2}} \\[3pt] \frac{|bic_{1} \cap bic_{2}|}{|bic_{1} \cup bic_{2}|}\end{array}\end{align*}

This score measures the average similarity between two sets of biclusters $G_{1}$ and $G_{2}$, where $|bic_{1} \cap bic_{2}|$ and $|bic_{1} \cup bic_{2}|$ are the elements in their intersection and union between two biclusters, respectively. Let $G_{1}$ and $G_{2}$ be the sets of true and predicted biclusters, respectively, then we have $score(G_{1}, G_{2})$ as the recovery score and $score(G_{2}, G_{1})$ as the relevance score.

### Simulated datasets

We implement a comparison of the OAEVOB’s performance with the following state-of-the-art algorithms.

- BP-EBA [26]
- Fast and accurate biclustering algorithm (RecBic) [28]
- Factor Analysis for BIcluster Acquisition (FABIA) [52]
- SSLB [53]
- ARBic [29]

The algorithms were executed with the default parameters specified in their respective papers. We compared the algorithms utilizing the gene coverage, number of biclusters, gene average, SDs computing relevance and recovery scores, and biclusters with a $p-value < 0.01$, which are the standard bases of comparison in the specialized literature. We employed Python v3.8 to implement OAEVOB (with the parameters described in Table 4) using a MacOS computer with a core i9 processor, having 10 cores, 64 GB of RAM, and 1TB of hard disk.

**Figure 8:**
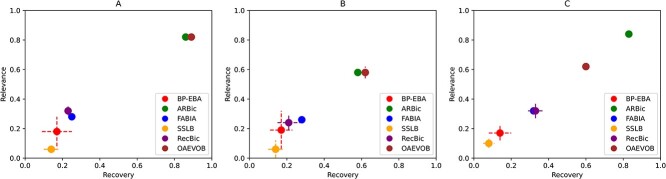
Relevance and recovery results in the SDs. A) Dataset with the implanted biclusters with an overlapping level of $50*50$. B) Dataset with the implanted biclusters with a noise level of $0.2$. C) Average across all SDs with implanted biclusters with different overlapping and level noise. OAEVOB obtained the greatest relevance and recovery scores in (A) and (B), while in (C), OAEVOB shows very competitive results with results of $0.62$ and $0.6$ in relevance and recovery scores, respectively, only outperformed by ARBic.

We created SD as described in [29]. We generated a $600*600$ data matrix sampled from a normal distribution $\mathcal{N}(0,1)$. The nine SDs generated contain different characteristics such as six trend-preserving biclusters, overlapping levels of $30*30$, $40*40$, $50*50$, without overlapping biclusters, a noise level of $0.1$, $0.2$, $0.3$, and without noisy biclusters. In the nine SDs generated, OAEVOB obtained an average of $0.62$ and $0.6$ in relevance and recovery scores, respectively. OAEVOB outperformed SSLB, BP-EBA, FABIA, and RecBic, obtaining very competitive results. ARBic is the only algorithm that outperformed OAEVOB. RecBic and FABIA obtained average scores lower than OAEVOB and ARBic. However, BP-EBA and SSLB obtained the lowest scores on average. The parameters used in these experiments are described in Table 5, and results in SDs are illustrated in Fig. 8.

**Table 5:** Parameters configuration in SDs generated with implanted biclusters. Values depict percentages of rows or columns

**Parameter**	**Simulated datasets**
Genes (rows)	[$0.166$, $0.166$]
Conditions (columns)	[$0.166$, $0.166$]

Furthermore, only OAEVOB is used on SDs generated with Python’s random number generator, using data distributions such as normal, Cauchy, and binomial. Table 6 provides information on the parameters used in this experiment. Table 7 shows the correlations that OAEVOB obtained in these SDs.

**Table 6:** Parameters configuration in SDs generated using statistical distributions. Values depict percentages of rows or columns

**Parameter**	**Normal**	**Cauchy**	**Binomial**
Genes (rows)	[0.1, 0.5]	[0.1, 0.5]	[0.1, 0.5]
Conditions (columns)	[0.01, 0.05]	[0.01, 0.05]	[0.01, 0.05]

**Table 7:** OAEVOB correlation results when applied to SDs generated using statistical distributions

**SD**	**Pearson corr.**	**Biweight midcorr.**	**Distance corr.**
Normal	0.4039 $\pm $ 0.0125	0.4017 $\pm $ 0.0097	0.41003 $\pm $ 0.0068
Cauchy	0.4051 $\pm $ 0.0086	0.3988 $\pm $ 0.0104	0.3925 $\pm $ 0.0291
Binomial	0.4019 $\pm $ 0.0247	0.3996 $\pm $ 0.0182	0.4015 $\pm $ 0.0158

Fitness normalized: $1$ is best and $0$ is worst. Average and $\pm $ standard deviation.

### Gene coverage

**Figure 9:**
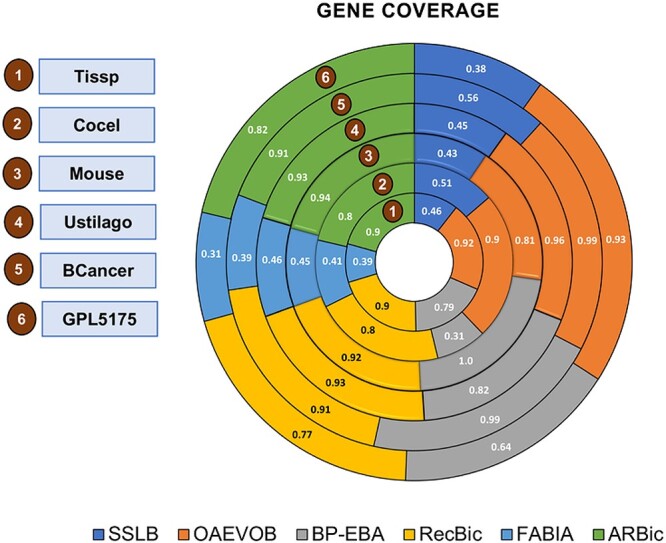
Gene coverage comparison in the six datasets ($1$ indicates that all genes were selected in any generation, and $0$ is the opposite, which is the worst value in this context). OAEVOB achieved a GeneCov greater than $0.8$ in all the datasets, and obtained the greatest GeneCov in Tissp, Cocel, Ustilago, BCancer, and GPL5175, only overcome by RecBic, BP-EBA, and ARBic, in Mouse. In contrast, SSLB and FABIA obtained the lowest GeneCov.

Examining each gene in the dataset is imperative to find novel gene relationships. It is crucial not to modify gene values based on predetermined criteria such as likelihood, neighborhood, or feature selection. Gene Coverage (GeneCov) analyzes when a gene appears in a bicluster of the current generation and is used to determine whether an algorithm succeeds in exploring the dataset. Enhancing gene coverage improves the analysis of different gene combinations and helps find biological functions. The JC computation allows OAEVOB to reach a GeneCov greater than $0.8$ across all datasets. Using SSLB, BP-EBA, RecBic, FABIA, ARBic, and OAEVOB, we calculated the GeneCov of Tissp, Cocel, Mouse, Ustilago, BCancer, and GPL5175, and the results are presented in Fig. 9. Additionally, Table 8 includes information regarding the parameters utilized in the GeneCov assessment.

**Table 8:** Parameters configuration in gene coverage. Values depict percentages of rows or columns

**Parameter**	**Genes (rows)**	**Conditions (columns)**
Tissp	[$0.01$, $0.04$]	[$0.04$, $0.1$]
Cocel	[$0.01$, $0.04$]	[$0.1$, $0.3$]
Mouse	[$0.05$, $0.15$]	[$0.001$, $0.003$]
Ustilago	[$0.025$, $0.1$]	[$0.04$, $0.1$]
BCancer	[$0.005$, $0.02$]	[$0.1$, $0.3$]
GPL5175	[$0.05$, $0.15$]	[$0.001$, $0.003$]

### Number of biclusters

Our work considers the number of biclusters (NB), which describes the number of biclusters obtained in an algorithm run. Potential future decision-making is ignored when the user’s preferences are not considered. Table 9 shows the results of NB.

**Table 9:** Summary of outcomes of the experiments performed to benchmark the state-of-the-art algorithms of the specialized literature

**Test**	**SSLB**	**BP-EBA**	**FABIA**	**OAEVOB**	**RecBic**	**ARBic**
Gene coverage	0.46 $\pm $ 0.05	0.75 $\pm $ 0.01	0.4 $\pm $ 0.03	**0.91 $\pm $ 0.01**	0.87 $\pm $ 0.01	0.88 $\pm $ 0.01
Number of biclusters	35.66 $\pm $ 0.16	51.33 $\pm $ 3.16	9.83 $\pm $ 1.41	**60 $\pm $ 0.0**	**60 $\pm $ 0.0**	**60 $\pm $ 0.0**
Average genes	21.76 $\pm $ 1.12	11.55 $\pm $ 1.21	6.61 $\pm $ 0.59	**140.14 $\pm $ 4.01**	91.89 $\pm $ 2.19	95.69 $\pm $ 1.87
Relevance	0.1 $\pm $ 0.03	0.17 $\pm $ 0.05	0.32 $\pm $ 0.01	0.62 $\pm $ 0.01	0.32 $\pm $ 0.05	**0.84 $\pm $ 0.0**
Recovery	0.08 $\pm $ 0.03	0.14 $\pm $ 0.06	0.32 $\pm $ 0.02	0.6 $\pm $ 0.01	0.33 $\pm $ 0.04	**0.83 $\pm $ 0.0**
Bic. $P$-value $< 0.01$	4.66 $\pm $ 0.2	6.29 $\pm $ 0.31	0.93 $\pm $ 0.19	**17.84 $\pm $ 0.47**	11.96 $\pm $ 0.55	12.24 $\pm $ 0.51

The highest result shows the best performance. The best result is in bold.

### Summary of the results

The comparison results in GeneCov, NB, gene average, relevance, recovery, and biclusters with a $p-value < 0.01$ are presented in Table 9.

## Gene enrichment analysis

The biological significance of OAEVOB’s results is analyzed using Gene Set Enrichment Analysis (GSEA), a method for identifying overrepresented gene classes associated with different phenotypes (different organism growth patterns or diseases) [54, 55]. The $P$-value is calculated by comparing the observed distribution to the null distribution, considering diagnostic/phenotypic labels, and adjus-ting for multiple hypothesis testing. GSEA evaluates the biological significance of the results achieved.

A strategy for tackling this analysis is to compute the Bonferroni Correction (BC) or BC adjusted (BCA). We also employed the False Discovery Rate (FDR), a reliable and widely accepted method, to address the false relationships detected by BCA. The enriched modules reported in this paper fulfilled BCA and FDR. We considered the sixty biclusters obtained by OAEVOB, ARBic, and RecBic. Concerning FABIA, BP-EBA, and SSLB, we considered their reported biclusters.

**Figure 10:**
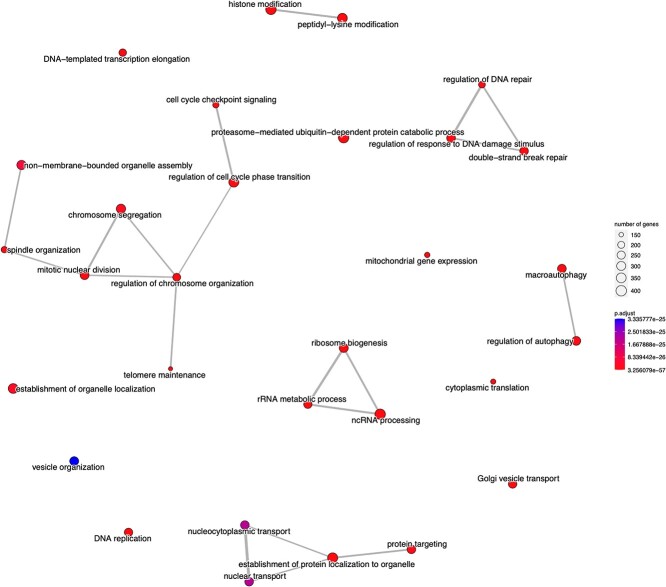
Module $6$, identified by OAEVOB in Cocel, has until $400$ genes involved in the biological functions and a $p-value < 0.01$. Many biological functions are linked (lines) between them, which indicates a strong relationship in the module.

**Figure 11:**
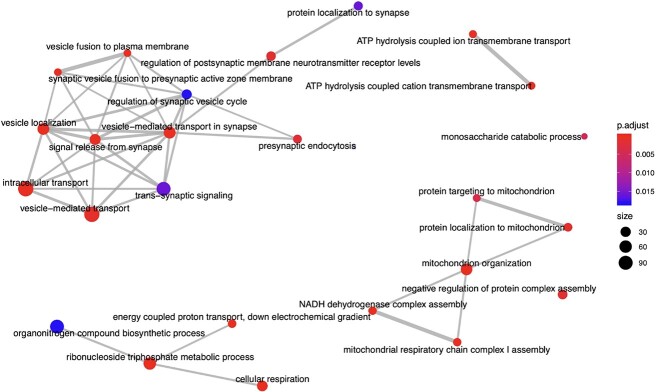
Module $60$, identified by OAEVOB in Mouse, obtaining a $p-value < 0.01$. Many biological functions are linked (lines) between them, which indicates a strong relationship in the module.

Due to Tissp’s dataset nature, we focus on identifying biological functions within a specific tissue. Concerning Cocel, we focused on identifying genes related to any cancer type. However, we will include other essential functions in this analysis, providing a comprehensive understanding of the datasets. Tables 10, 11, and 12 show the enriched modules identified by OAEVOB in Tissp, Cocel, and Mouse, respectively, with colors assigned for easier visualization of biological functions within the modules. Table 11 shows $16$ enriched modules with testis cancer; therefore, the color assigned to this biological function is the most frequent. In Table 12, no color is repeated, indicating that no biological function enriches more than one module. In Figs 10 and 11, we used the enrichplot R library to illustrate the enriched modules identified by OAEVOB in Cocel and Mouse, respectively [56].

**Table 10:** Biological function analysis in Tissp using OAEVOB. The identified biological functions of each module are highlighted in distinct colors to visualize the ones that appear more frequently quickly. In cases where the module has no enriched biological function in the characteristic indicated in the column, a ‘-’ is displayed

**Module**	**Tissue-specific**	**Biological function**	**Molecular function**
2	Kidney	Stress response	-
4	Intestine	-	-
5	Endometrium	-	-
8	-	Angiogenesis	-
9	-	Growth regulation	Helicase
12	-	mRNA processing	-
14	-	Autophagy	-
14	-	DNA damage, repair	Chaperone
17	Epididymis	Host-virus interaction	-
23	Kidney	Fertilization	Antioxidant
27	Tongue	-	Oxidoreductase
28	Tongue	Inflammatory response	RNA-binding
30	Intestine	-	-
37	Tongue	Neurogenesis	Hydrolase
42	Tongue	mRNA splicing	-

**Table 11:** Biological function analysis in Cocel using OAEVOB. The identified biological functions of each module are highlighted in distinct colors to visualize the ones that appear more frequently quickly. In cases where the module has no enriched biological function in the characteristic indicated in the column, a ‘-’ is displayed

**Module**	**Cancer**	**Protein**	**Biological function**
1	Testis	-	-
2	Testis	Cardiovascular disease	-
4	Testis	Plasma proteins	-
5	-	-	Adaptive immunity
6	Testis	Cancer-related genes	Adaptive immunity
10	Testis	-	-
13	-	Cancer-related genes	Immunity
14	-	-	Adaptive immunity
16	Testis	-	Adaptive immunity
17	-	Cancer-related genes	-
18	Testis	-	-
19	Testis	-	-
21	Testis	Predicted secreted protein	-
23	Testis	Predicted intracellular protein	-
28	Testis	-	-
39	Testis	Disease related genes	Immunity
42	Testis	Cancer-related genes	Adaptive immunity
46	Testis	Cancer-related genes	-
47	-	Cancer-related genes	-
48	Testis	FDA drug targets	Adaptive immunity
50	Testis	-	-

**Table 12:** Biological function analysis in Mouse using OAEVOB. The identified biological functions of each module are highlighted in distinct colors to visualize the ones that appear more frequently quickly. In cases where the module has no enriched biological function in the characteristic indicated in the column, a ‘-’ is displayed

**Module**	**Biological function**	**Molecular function**
35	Regulation of kidney development	ERK1, ERK2 cascade
45	Renal-tubule development	MAPK cascade
58	Epithelial cell differentiation	Signaling pathway
60	Cellular respiration	Mitochondrial respiratory

Tables 13, 14, and 15 show the enriched modules identified by RecBic in Tissp, BP-EBA in Cocel, and SSLB in Cocel, respectively.

**Table 13:** Biological function analysis in Tissp using RecBic. In cases where the module has no enriched biological function in the characteristic indicated in the column, a ‘-’ is displayed

**Module**	**Tissue-specific**	**Biological function**	**Protein**
1	-	Heart contraction	Protein polyubiquitination
4	Epidermis	Regulation of membrane potential	Protein-coupled receptor signaling pathway
8	Epidermis	Epithelial cell proliferation	-
9	-	Regulation of blood circulation	MAPK cascade
21	-	Cardiac and striated muscle tissue development	Activation of protein kinase activity
25	Epidermis	Heart process	-

**Table 14:** Biological function analysis in Cocel using BP-EBA. In cases where the module has no enriched biological function in the characteristic indicated in the column, a ‘-’ is displayed

**Module**	**Biological function**	**Protein**
4	Myosin phosphatase	Serine/threonine phosphatase
6	RNA splicing	Ubiquitin conjugating enzyme
15	Phosphatase activity	-
24	AMP metabolic process	-

**Table 15:** Biological function analysis in Cocel using SSLB. In cases where the module has no enriched biological function in the characteristic indicated in the column, a ‘-’ is displayed

**Module**	**Biological function**	**Molecular function**
1	Golgi vesicle transport	Organelle fusion
2	Regulation of binding	-
36	Regulation of apoptotic signaling	Ras signal transduction

**Figure 12:**
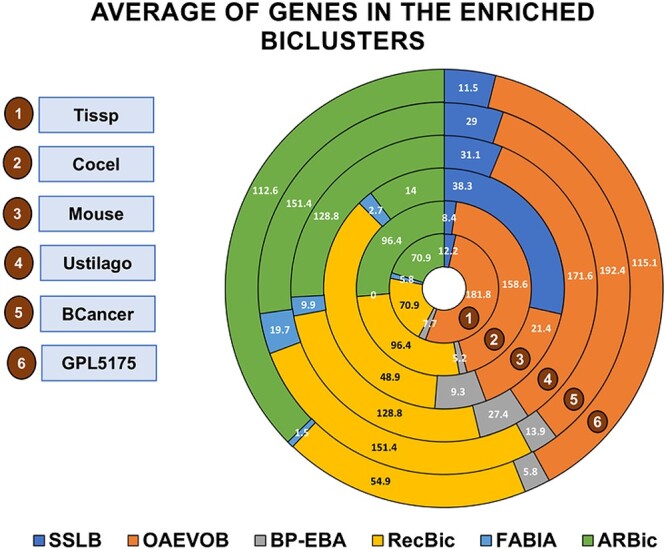
OAEVOB obtained the highest average number of genes in Tissp, Cocel, Ustilago, BCancer, and GPL5175. On the other hand, RecBic had the highest result in Mouse. BP-EBA and FABIA had the lowest average number of genes across all datasets.

**Figure 13:**
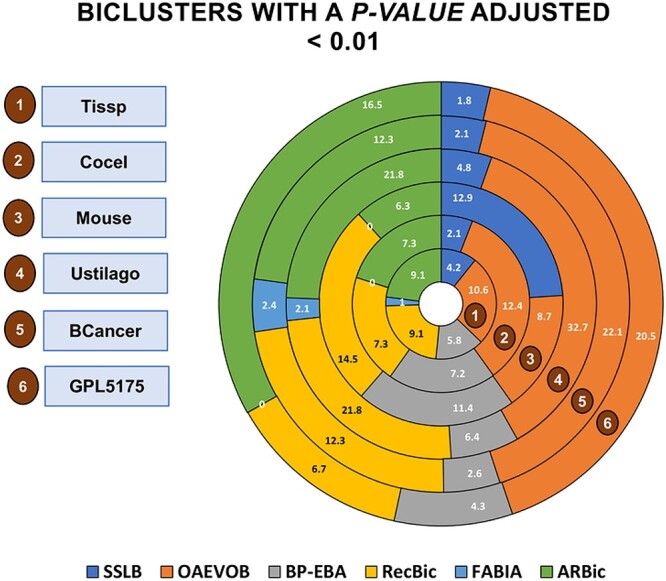
OAVEOB obtained the greatest number of biclusters with a $p-value < 0.01$ in Tissp, Cocel, Ustilago, BCancer, and GPL5175. RecBic reported the greatest result for Mouse. Conversely, FABIA obtained the lowest number of biclusters across all datasets.

Figure 12 shows the average number of genes included in the found modules. Figure 13 shows the number of biclusters with a $p-value < 0.01$.

We include a comprehensive analysis of the enriched biclusters found by OAEVOB in Tissp, Cocel, and Mouse. In Tissp, we found relevant genes related to tissue-specific in module $2$. $ABCC3$ and $ABCC4$ are crucial for detoxification and drug metabolism in the Kidney, involved in the transport of organic anions across cellular membranes, and their role in neurotransmitter transport and drug excretion, respectively [57]. In module $23$, the main functions are related to metabolism and detoxification in genes $CYP1A1$ and $AGMAT$, which are involved in the detoxification of harmful compounds and the regulation of lipid metabolism, with key roles in the liver and kidney. In module $28$, the genes $BCL9$, $ZEB2$, and $PITX3$ have relevance in cellular development and differentiation and are involved in cellular signaling pathways that regulate development, differentiation, and survival, particularly in tissues like the nervous system, lungs, and muscles.

In Cocel’s module $1$, we found biological functions in genes related to metastasis and migration in cancer. $SMAD2$ is part of the $TGF-\beta $ signaling pathway and is involved in regulating cell migration, differentiation, and metastasis [58]. In cancer, the $TGF-\beta $ pathway can switch from a tumor suppressor to a pro-metastatic role. In addition, upregulation of $SNHG15$ is linked to poor prognosis in cancers like colorectal, breast, and gastric cancer, promoting proliferation and metastasis [59]. Mutations in $GALNT12$ have been linked to colorectal cancer due to altered glycosylation patterns affecting cell signaling [60]. In module $2$, functions related to DNA damage response and repair are found. Mutations in $ATM$ contribute to cancer development by impairing the cell’s ability to repair DNA [61]. Mutations in $MRE11$ can impair DNA repair mechanisms and lead to genomic instability [62]. Furthermore, the cancer relevance of overexpression of $ABCC10$ is associated with resistance to chemotherapy, particularly in breast, lung, and ovarian cancers. $ABI2$ is implicated in processes related to metastasis in lung and breast cancer, by influencing cell motility and invasion. In module $6$, disruption of lysosomal function in $BLOC1S5$ is associated with tumor growth, metastasis, and resistance to therapy. Altered lipid metabolism, often driven by $INSIG2$, supports rapid cell proliferation. Dysregulation of $XIST$ can alter epigenetic landscapes, leading to tumor progression, particularly in sex-specific cancers.

In module $21$, $ALDH1B1$ is often used as a marker for cancer stem cells, and its expression is associated with poor prognosis, particularly in liver and colon cancer. $BCL11B$ is implicated in the development of T-cell acute lymphoblastic leukemia (T-ALL) and other lymphomas, and its expression may influence tumor progression. In module $39$, $CEP164$ is a protein involved in the formation and function of the centrosome, which is critical for proper cell division. Alterations in $CEP164$ are associated with defects in mitosis, leading to aneuploidy, a common feature in cancer cells. In module $42$, dysregulation of $MAP2K2$ is common in melanomas, promoting uncontrolled cell proliferation and resistance to cell death. Alterations in $SMAD9$ function can lead to the disruption of $TGF-\beta $ signaling, contributing to cancer progression by promoting cell proliferation and inhibiting apoptosis. $TP53BP1$ is crucial for maintaining genomic stability. Mutations or deletions of $TP53BP1$ may lead to defective DNA repair, contributing to cancer development and resistance to therapy. These grouped genes reflect a wide range of functions essential for regular cellular processes and their dysregulation or mutations are associated with a variety of cancers, often contributing to cell cycle deregulation ($CCNB2, MCM7, CDK20$), metastasis and migration ($PLXND1, SMAD2$), resistance to apoptosis ($BMF, CASP6, TRIM66$), DNA damage repair, and genomic instability. These genes may serve as potential biomarkers for cancer diagnosis, prognosis, and therapy.

Module $35$ of Mouse contains a broad range of molecules involved in brain function, neuroplasticity, and learning. $Synpr$ and $Capsl$ are implicated in synaptic vesicle trafficking and neurotransmitter release, playing key roles in synaptic plasticity and learning [63]. $Netrin1$ is involved in axon guidance and synapse formation, which are crucial for brain development and function [64]. Module $45$ has biological functions related to neurotransmitter synthesis and signaling. $Th$ is essential for the synthesis of dopamine, a critical neurotransmitter involved in motor control, reward processing, and cognitive functions. $Ddc$ is involved in the conversion of $L-DOPA$ to dopamine and serotonin, impacting mood regulation and motor function. $Grin1$ and $Grin3a$ encode subunits of the $NMDA$ receptor, which is critical for synaptic plasticity, learning, and memory formation.

## Discussion

In this article, we develop a novel algorithm for identifying biclustering in gene expression matrices. We use six datasets extensively studied in the literature for pattern recognition in computational algorithms, as there is no gold standard for benchmarking biclustering algorithms. The Tissp dataset has been analyzed in [65–68], Cocel has been used in [69, 70], Mouse in [71–74], Ustilago in [38], BCancer in [39], and GPL5175 in [29]. These datasets have different structures and provide valuable information from different sequencing gene expression data, making it more challenging to identify diverse patterns compared to SDs. Analyzing real datasets is more challenging but yields more precise results.

It was identified that all datasets analyzed with OAEVOB obtained biclusters with the highest correlations, with a fitness greater than $0.52$ and a MI greater than $0.99$ (Table 2). These results demonstrate that the correlations and MI achieved by OAEVOB in these biclusters are statistically significant, surpassing the threshold of 0.5.

In Fig. 8, we show that OAEVOB outperforms ARBic, RecBic, SSLB, BP-EBA, and FABIA in relevance and recovery scores in two SDs with implanted biclusters with an overlapping level of $50*50$ and a noise level of $0.2$, showing that OAEVOB is robust to noise and overlapping. OAEVOB obtained very competitive results with average recovery and relevance scores of $0.6$ and $0.62$, respectively. OAEVOB outperformed RecBic, SSLB, BP-EBA, and FABIA on average, only outperformed by ARBic.

Table 7 shows that OAEVOB obtained a correlation of approximately $0.4$ in SDs generated using normal, Cauchy, and binomial distributions, which is not statistically significant (less than $0.5$). This suggests that OAEVOB obtained the expected results, as no significant relationship was expected in these SDs.

In Tissp, Cocel, Ustilago, BCancer, and GPL5175, OAEVOB obtained higher GeneCov than all state-of-the-art algorithms (Fig. 9). In Mouse, BP-EBA achieved the highest GeneCov. RecBic and ARBic outperformed BP-EBA, FABIA, and SSLB on Tissp, Cocel, Ustilago, and GPL5175. FABIA and SSLB showed the poorest performance in GeneCov. OAEVOB’s exploration is notably more extensive, indicating that all potential relationships were included and analyzed at some point.

The user specifies the NB value in OAEVOB, RecBic, and BP-EBA. OAEVOB, ARBic, and RecBic fulfilled the user’s request for all six datasets (Table 9). BP-EBA retained less than sixty biclusters, as it includes a final step to filter the NB, thereby demonstrating its limitations. SSLB and FABIA assign the NB internally but do not meet the user’s minimum NB requirement.

Table 9 shows that OAEVOB outperformed the state-of-the-art algorithms in GeneCov, average genes, and biclusters with a $p-value < 0.01$, and obtained very competitive results in relevance and recovery scores, which are classical evaluations in the specialized literature. This suggests that OAEVOB is highly competitive in analyzing gene expression data from diverse sources.

Regarding the GSEA results, OAEVOB identified genes related to specific tissues such as the tongue, kidney, intestine, endometrium, and epididymis in Tissp in the enriched modules (Table 10). Therefore, OAEVOB effectively distinguished and grouped tissue-specific genes. OAEVOB also detected biological functions related to stress response, neurogenesis, RNA binding, and host–virus interaction. The enriched modules in Tissp contain an average of $181$ genes, and in most biclusters, the adjusted $P$-value is less than $0.01$ (Fig. 13).

In Cocel (Table 11), we focused on identifying essential cancer-related biological functions. OAEVOB identified testis cancer genes in seventeen enriched modules, distinguishing and grouping cancer-related genes. Biological functions were also found in cancer-related genes, cardiovascular disease, and immunity. Enriched modules include an average of $158$ genes, with most biclusters having a $p-value < 0.01$. In Fig. 10, the enrichplot R library is used to illustrate the enriched module $6$ identified by OAEVOB.

In Mouse, OAEVOB identified functions that regulate kidney development, epithelial cell differen-tiation, cellular respiration, and signaling pathways (Table 12). On average, these biclusters contain $21$ genes, with an adjusted $p-value < 0.01$ in two biclusters. Figure 11 shows an enriched bicluster.

In Tissp, RecBic detected genes epidermis (three modules) and heart-related biological functions (Table 13). Most enriched modules obtained an adjusted $p-value < 0.01$. In comparison, OAEVOB identified 10 enriched modules with specific tissues. In Cocel, RecBic found enriched modules related to the biological functions of cardiovascular disease and cancer-related genes, containing an average of $96$ genes. None of the modules found by RecBic are related to a specific cancer type. In Mouse, the modules are primarily involved in RNA splicing and brain-related functions. Compared to OAEVOB, SSLB, BP-EBA, and FABIA, these modules have the highest average number of genes and modules with an adjusted $p-value < 0.01$. ARBic identified biological functions unrelated to specific tissues and any cancer type in Tissp and Cocel, respectively. ARBic detected enriched modules related to transduction and signaling in Mouse.

In Tissp, BP-EBA found biological functions associated with tissue migration and protein depoly-merization unrelated to any specific tissue. Four modules contained two genes and an adjusted $p-value < 0.01$. In Cocel, the enriched modules are related to RNA splicing and phosphatase activity (Table 14), unrelated to any cancer type. The modules contained the fewest genes, and only three modules obtained an adjusted $p-value < 0.01$. In Mouse, aggressive behavior and tumor necrosis were the biological functions found, and most obtained an adjusted $p-value < 0.01$.

In Tissp, SSLB and ARBic identified biological functions unrelated to specific tissues, such as the regulation of autophagy and peptide hormones. In Cocel, SSLB and ARBic did not identify any cancer type (Table 15). Thirteen enriched modules, mainly related to brain signaling, were detected in Mouse (surpassed only by RecBic).

FABIA detected no enriched modules in Cocel and only one in Tissp and Mouse, unrelated to any specific tissue or cancer.

## Conclusions and future work

In OAEVOB’s initial exploration, biclusters with many genes are identified, comprising highly correlated genes of substantial size. OAEVOB selects a specific seed for each bicluster. The JC computation accurately detects and discards similar biclusters to improve the uniqueness and quality of resulting biclusters. Additionally, adjusting the $\eta c$ and mutation probability values enhances the biclusters’ fitness, enabling OAEVOB to find diverse solutions when necessary and exploit biclusters with high correlations.

In [23], it is discussed how $ACV$ can detect biclusters’ scale, shift, and scale-shift patterns and how $ACV$ and $VE^{t}$ correlate highly with gene ontology. We compute $ACV$ using similarity measurements such as Pearson correlation, Biweight midcorrelation, distance correlation, and MI to strengthen the detection of scale and shifting patterns. This is useful for analyzing complex biological systems, where elements have multiple functions and are functionally diverse, making them more likely to interact nonlinearly.

Our novel approach includes an online-adjustment component that changes dynamically to create offspring. Online-adjustment balances offspring diver-sity based on fitness, allowing OAEVOB to identify high-quality solutions while maintaining the necessary randomness for exploration. If mutation and offspring diversity are not adequately regulated, the algorithm may continue to explore excessively. This can result in slower convergence and unnecessary computations, especially in large and complex search spaces, such as those encountered in RNA-seq datasets. A lower mutation rate and reduced offspring diversity help the algorithm fine-tune solutions and converge to the optimal solution. Higher mutation rates and different offspring promote diversity, preventing stagnation and avoiding suboptimal areas. This balance sustains genetic diversity and enhances the algorithm’s robustness in complex fitness landscapes, enhancing the overall search process. Using MIXRNG helps avoid the bias imposed by employing a single RNG.

OAEVOB efficiently finds significant modules in gene expression data. The GSEA results show that OAEVOB identifies many modules containing genes related to cancer types and specific tissues. OAEVOB found enriched modules with the highest number of genes in Tissp, Cocel, Ustilago, BCancer, and GPL5175. In Mouse, it is only outperformed by RecBic and SSLB (Fig. 12). This is essential for identifying more genes associated with specific cancer types and tissues that were previously unknown and potentially advancing our understanding of cancer biology. Additionally, OAEVOB outperforms the state-of-the-art algorithms in Tissp, Cocel, Ustilago, BCancer, and GPL5175, as it identifies the highest number of enriched modules with an adjusted $p-value < 0.01$ (Fig. 13).

We conclude that OAEVOB highly differentiates genes associated with specific tissues in Tissp and cancer-related genes in Cocel; however, RecBic, ARBic, SSLB, BP-EBA, and FABIA cannot equally differentiate these genes.

Additionally, OAEVOB identifies enriched narrow biclusters, which consist of a small number of conditions, and also broader biclusters, which include many conditions. While RecBic is particularly effective at finding high-quality narrow biclusters [29], and ARBic specializes in identifying high-quality broader biclusters, OAEVOB shows superior performance than RecBic and ARBic in both bicluster sizes. OAEVOB outperforms RecBic in datasets with less and more than $500$ columns. This indicates that OAEVOB also surpasses ARBic in datasets containing less than $500$ columns. OAEVOB’s performance surpassed RecBic, ARBic, BP-EBA, FABIA, and SSLB in SDs to find noisy and overlapped biclusters obtaining higher recovery and relevance scores. Most biclustering algorithms, in general, perform comparably or exhibit inferior results compared to random approaches on human datasets due to a high incidence of false positives [75]. Therefore, identifying concealed relationships and patterns within human datasets is more challenging, as these datasets tend to be more complex than simulated ones. OAEVOB demonstrates superiority in identifying complex enriched narrow and broader biclusters in our comparison. Furthermore, OAEVOB’s complexity remains highly competitive, utilizing Pearson and biweight midcorrelation with a complexity of $O(n^{2})$.

Exploring different datasets in structure is critical to improving OAEVOB’s performance. Implementing a parallelized architecture to run OAEVOB on a GPU might significantly reduce computational time. Our future work will analyze unique modules of genes that are not predicted by state-of-the-art algorithms. Future work also includes analyzing overlapping genes to identify novel relationships and patterns, performing various crossover types, and developing a multi-objective algorithm with external archivers to identify conflicting similarity measurements.

Our paper provides a gateway for developing novel biclustering algorithms. Therefore, bioinformatics can use OAEVOB to discover novel and significant biological insights from gene expression data, making it a valuable tool for the field.

**Key Points**

- OAEVOB, a novel biclustering algorithm.
- OAEVOB detects significant modules in microarray and RNA-seq datasets.
- OAEVOB identifies significant modules in scRNA-seq.
- Online-adjustment helps form meaningful biclusters.
- OAEVOB outperforms ARBic and RecBic in finding significant biclusters.

## References

[1] de Sousa JS , Gomes LDCT, Bezerra GB. et al. An immune-evolutionary algorithm for multiple rearrangements of gene expression data. Genet Program Evolvable Mach 2004;5:157–79. 10.1023/B:GENP.0000023686.59617.57.

[2] Orphanides G , Reinberg D. A unified theory of gene expression. Cell 2002;108:439–51. 10.1016/S0092-8674(02)00655-4. 11909516

[3] Clamp M , Fry B, Kamal M. et al. Distinguishing protein-coding and noncoding genes in the human genome. Proc Natl Acad Sci USA 2007;104:19428–33. 10.1073/pnas.0709013104. 18040051 PMC2148306

[4] Tupler R , Perini G, Green M. Expressing the human genome. Nature 2001;409:832–3. 10.1038/35057011. 11237001

[5] Berg JM , Tymoczko JL, Gatto JGJ. et al. Biochemistry, 5th edn. New York: W H Freeman, 2002.

[6] Joseph F . Quantitative Human Physiology: An Introduction 2nd edn. Academic Press, 2017.

[7] Katahira J . Nuclear export of messenger RNA. Genes (Basel) 2015;6:163–84. 10.3390/genes6020163. 25836925 PMC4488659

[8] Jinzhi L . Systems Biology: Modeling, Analysis, and Simulation. Springer International Publishing, 2021.

[9] Lind C , Åqvist J. Principles of start codon recognition in eukaryotic translation initiation. Nucleic Acids Res 2016;44:8425–32. 10.1093/nar/gkw534. 27280974 PMC5041461

[10] Cooper G . The Cell: A Molecular Approach. Sunderland, 2000.

[11] Nehete JY , Bhambar RS, Narkhede MR. et al. Natural proteins: Sources, isolation, characterization and applications. Pharmacogn Rev 2013;7:107–16. 10.4103/0973-7847.120508. 24347918 PMC3841988

[12] Barrett T , Evangelista C. NCBI GEO: archive for functional genomics data sets–update. Nucleic Acids Res 2013;41:D991–5. 23193258 10.1093/nar/gks1193 PMC3531084

[13] Moretto M , Dierckxsens N. COLOMBOS v3.0: leveraging gene expression compendia for cross-species analyses. Nucleic Acids Res 2016;44:D620–3. 10.1093/nar/gkv1251. 26586805 PMC4702885

[14] Su AI , Wiltshire T, Batalov S. et al. A gene atlas of the mouse and human protein-encoding transcriptomes. Proc Natl Acad Sci USA 2004;101:6062–7. 10.1073/pnas.0400782101. 15075390 PMC395923

[15] Nusinow DP , Szpyt J, Ghandi M. et al. Quantitative proteomics of the cancer cell line encyclopedia. Cell 2020;180:387–402.e16. 10.1016/j.cell.2019.12.023. 31978347 PMC7339254

[16] Kanehisa M , Goto S. Kegg: Kyoto encyclopedia of genes and genomes. Nucleic Acids Res 2000;28:27–30. 10.1093/nar/28.1.27. 10592173 PMC102409

[17] Palasca O , Santos A, Stolte C. et al. Tissues 2.0: an integrative web resource on mammalian tissue expression. Database (Oxford) 2018;2018:bay003. 10.1093/database/bay003. PMC5808782 29617745

[18] Patryk O , Moshe S, Xiuzhen H. et al. Ebic: an evolutionary-based parallel biclustering algorithm for pattern discovery. Bioinformatics 2018;34:3719–26. 29790909 10.1093/bioinformatics/bty401 PMC6198864

[19] Ons M , Emna A, Hend B. et al. Bobea: a bi-objective biclustering evolutionary algorithm for genome-wide association analysis. In: Proceedings of the Genetic and Evolutionary Computation Conference (GECCO), pp. 344–7. New York, NY, USA: ACM, 2022.

[20] Ons M , Wassim A, Hend B. et al. Evolutionary local search algorithm for the biclustering of gene expression data based on biological knowledge. Appl Soft Comput 2021;104:107177. 10.1016/j.asoc.2021.107177.

[21] Nicholls K , Wallace C. Comparison of sparse biclustering algorithms for gene expression datasets. Brief Bioinform 2021;22:bbab140. 10.1093/bib/bbab140. PMC8574648 33951731

[22] Cheng Y , Church GM. Biclustering of expression data. In: Proceedings of the International Conference on Intelligent Systems for Molecular Biology (ISMB). AAAI Press, vol. 8, pp. 93–103, 2000. 10977070

[23] Pontes B , Girldez R, Aguilar-Ruiz JS. Quality measures for gene expression biclusters. PloS One 2015;10:e0115497. 10.1371/journal.pone.0115497. 25763839 PMC4357449

[24] Ons M , Wassim A, Hend B. et al. Evolutionary local search algorithm for biclustering of gene expression data based on biological knowledge. Appl Soft Comput 2021;104:107177.

[25] Maâtouk O , Ayadi W, Bouziri H. et al. Evolutionary biclustering algorithms: an experimental study on microarray data. Soft Comput 2019;23:7671–97.

[26] Huang Q , Huang X, Kong Z. et al. Bi-phase evolutionary searching for biclusters in gene expression data. IEEE Trans Evol Comput 2019;23:803–14. 10.1109/TEVC.2018.2884521.

[27] Juan X , Anjun M, Yu Z. et al. QUBIC2: a novel and robust biclustering algorithm for analyses and interpretation of large-scale RNA-seq data. Bioinformatics 2020;36:1143–9. 31503285 10.1093/bioinformatics/btz692 PMC8215922

[28] Liu X , Li D, Liu J. et al. RecBic: a fast and accurate algorithm recognizing trend-preserving biclusters. Bioinformatics 2020;36:5054–60. 10.1093/bioinformatics/btaa630. 32653907

[29] Xiangyu L , Ting Y, Xiaoyu Z. et al. ARBic: an all-round biclustering algorithm for analyzing gene expression data. NAR Genom Bioinform 2023;5:lqad009. 36733402 10.1093/nargab/lqad009 PMC9887595

[30] Sriwastava BK , Halder AK, Basu S. et al. RUBic: rapid unsupervised biclustering. BMC Bioinform 2023;24:435. 10.1186/s12859-023-05534-3. PMC10655409 37974081

[31] Qiyiwen Z , Changgee C, Qi L. Robust knowledge-guided biclustering for multi-omics data. Brief Bioinform 2024;25. 10.1093/bib/bbad446. PMC10701104 38058188

[32] Jianjun S , Qinghua H. Two stages biclustering with three populations. Biomed Signal Process Control 2023;79:104182. 10.1016/j.bspc.2022.104182.

[33] Bumgarner R . Overview of DNA microarrays: types, applications, and their future. Curr Protoc Mol Biolpage Unit 2013;101:1. 10.1002/0471142727.mb2201s101. PMC4011503 23288464

[34] Stark R , Grzelak M, Hadfield J. RNA sequencing: the teenage years. Nat Rev Genet 2019;20:631–56. 10.1038/s41576-019-0150-2. 31341269

[35] Jovic D , Liang X, Zeng H. et al. Single-cell RNA sequencing technologies and applications: a brief overview. Clin Transl Med 2022;12:e694. 10.1002/ctm2.694. 35352511 PMC8964935

[36] Schlicker A , Beran G, Chresta CM. et al. Subtypes of primary colorectal tumors correlate with response to targeted treatment in colorectal cell lines. BMC Med Genom 2012;5:66. 10.1186/1755-8794-5-66. PMC3543849 23272949

[37] Islam S , Zeisel A, Joost S. et al. Quantitative single-cell RNA-seq with unique molecular identifiers. Nat Methods 2014;11:163–6. 10.1038/nmeth.2772. 24363023

[38] Soberanes-Gutiérrez CV , Castillo-Jiménez A, Pérez-Rueda E. et al. Construction and analysis of gene co-expression network in the pathogenic fungus Ustilago maydis. Front Microbiol 2022;13:1048694. 10.3389/fmicb.2022.1048694. 36569046 PMC9767968

[39] Mares-Quiñones MD , Galán-Vásquez E, Pérez-Rueda E. et al. Identification of modules and key genes associated with breast cancer subtypes through network analysis. Sci Rep 2024;14:12350. 10.1038/s41598-024-61908-4. 38811600 PMC11137066

[40] Nepomuceno JA , Troncoso A, Aguilar-Ruiz JS. A Hybrid Metaheuristic for Biclustering Based on Scatter Search and Genetic Algorithms. In: Pattern Recognition in Bioinformatics, vol. 5780 of *Lecture Notes in Computer Science*. Berlin, Heidelberg: Springer, 2009. 10.1007/978-3-642-04031-3_18.

[41] Knuth DE . The Art of Computer Programming3rd edn. Boston, MA: Addison Wesley, 1997.

[42] Draper D , Erdong G. The Practical Scope of the Central Limit Theorem, 2021.

[43] Aldana-Bobadilla E , Kuri-Morales A. Unsupervised Classifier Based on Heuristic Optimization and Maximum Entropy Principle. In: Advances in Intelligent Systems and Computing, vol. 227. Heidelberg: Springer, 2013. 10.1007/978-3-319-01128-8_2.

[44] Kalyanmoy D , Agrawal RB. Simulated binary crossover for continuous search space. Complex Syst 1995;9.

[45] Benesty J , Chen J, Huang Y. On the importance of the Pearson correlation coefficient in noise reduction. IEEE Trans Audio Speech Lang Process 2008;16:757–65. 10.1109/TASL.2008.919072.

[46] Zheng CH , Yuan L, Sha W. et al. Gene differential coexpression analysis based on biweight correlation and maximum clique. BMC Bioinform 2014;15:S3. 10.1186/1471-2105-15-S15-S3. PMC4271563 25474074

[47] Székely GJ , Maria LR. Brownian distance covariance. Ann Appl Stat 2009;3:1236–65. 10.1214/09-AOAS312. PMC2889501 20574547

[48] Duncan TE . On the calculation of mutual information. SIAM J Appl Math 1970;19:215–20. 10.1137/0119020.

[49] Fagerland MW , Sandvik L. The Wilcoxon-Mann-Whitney test under scrutiny. Stat Med 2009;28:1487–97. 10.1002/sim.3561. 19247980

[50] Martínez-Murcia FJ , Górriz JM, Ramírez J. et al. Computer aided diagnosis tool for Alzheimer’s disease based on Mann-Whitney-Wilcoxon U-test. Expert Systems with Applications 2012;39:9676–85. 10.1016/j.eswa.2012.02.153.

[51] Prelić A , Bleuler S, Zimmermann P. et al. A systematic comparison and evaluation of biclustering methods for gene expression data. Bioinformatics 2006;22:1122–9. 10.1093/bioinformatics/btl060. 16500941

[52] Hochreiter S , Bodenhofer U, Heusel M. et al. FABIA: Factor Analysis for Bicluster Acquisition. Bioinformatics 2010;26:1520–7. 10.1093/bioinformatics/btq227. 20418340 PMC2881408

[53] Moran GE , Rocková V, George EI. Spike-and-slab lasso biclustering. Ann Appl Stat 2021;15:148–73. 10.1214/20-AOAS1385.

[54] Subramanian A , Tamayo P, Mootha VK. et al. Gene set enrichment analysis: a knowledge-based approach for interpreting genome-wide expression profiles. Proc Natl Acad Sci U S A 2005;102:15545–50. 10.1073/pnas.0506580102. 16199517 PMC1239896

[55] Sherman BT , Hao M, Qiu J. et al. DAVID: a web server for functional enrichment analysis and functional annotation of gene lists (2021 update). Nucleic Acids Res 2022;50:W216–21. 10.1093/nar/gkac194. 35325185 PMC9252805

[56] Yu G . enrichplot: visualization of functional enrichment result. R package version 1.26.5. 2024.

[57] Zhao Y , Lu H, Yan A. et al. ABCC3 as a marker for multidrug resistance in non-small cell lung cancer. Sci Rep 2013;3:3120. 10.1038/srep03120. 24176985 PMC3814586

[58] Girolami I , Veronese N, Smith L. et al. The activation status of the TGF-$\beta $ transducer SMAD2 is associated with a reduced survival in gastrointestinal cancers: a systematic review and meta-analysis. Int J Mol Sci 2019;20:3831. 10.3390/ijms20153831. 31387321 PMC6695973

[59] Chen C , Feng Y, Wang J. et al. Long non-coding RNA SNHG15 in various cancers: a meta and bioinformatic analysis. BMC Cancer 2020;20:1156. 10.1186/s12885-020-07649-9. 33243205 PMC7690101

[60] Evans DR , Venkitachalam S, Revoredo L. et al. Evidence for GALNT12 as a moderate penetrance gene for colorectal cancer. Hum Mutat 2018;39:1092–101. 10.1002/humu.23549. 29749045 PMC6043371

[61] Stucci LS , Internò V, Tucci M. et al. The ATM gene in breast cancer: its relevance in clinical practice. Genes 2021;12:727. 10.3390/genes12050727. 34068084 PMC8152746

[62] Cho MG , Kumar RJ, Lin CC. et al. MRE11 liberates cGAS from nucleosome sequestration during tumorigenesis. Nature 2024;625:585–92. 10.1038/s41586-023-06889-6. 38200309 PMC10794148

[63] Matson KJE , Russ DE, Kathe C. et al. Single cell atlas of spinal cord injury in mice reveals a pro-regenerative signature in spinocerebellar neurons. Nat Commun 2022;13:5628. 10.1038/s41467-022-33184-1. 36163250 PMC9513082

[64] Cassier PA , Navaridas R, Bellina M. et al. Netrin-1 blockade inhibits tumour growth and emt features in endometrial cancer. Nature 2023;620:409–16. 10.1038/s41586-023-06367-z. 37532934 PMC10412451

[65] Carmona-Saez P , Pascual-Marqui RD, Tirado F. et al. Biclustering of gene expression data by non-smooth non-negative matrix factorization. BMC Bioinform 2006;7:78. 10.1186/1471-2105-7-78. PMC1434777 16503973

[66] Wong M , Mutch D, McNicholas P. Two-way learning with one-way supervision for gene expression data. BMC Bioinform 2017;18:150. 10.1186/s12859-017-1564-5. PMC5336648 28257645

[67] Lahti L , Knuuttila JE, Kaski S. Global modeling of transcriptional responses in interaction networks. Bioinformatics 2010;26:2713–20. 10.1093/bioinformatics/btq500. 20813878

[68] Atushi N , Seiya I, Rui Y. et al. Model-free unsupervised gene set screening based on information enrichment in expression profiles. Bioinformatics 2010;26:3090–7. 10.1093/bioinformatics/btq592. 20959379

[69] Sha C , Wennan C, Changlin W. et al. Bi-Clustering Based Biological and Clinical Characterization of Colorectal Cancer in Complementary to CMS Classification. Cold Spring Harbor Laboratory, bioRxiv, 2018. 10.1101/508275.

[70] Wennan C , Changlin W, Yong Z. et al. Supervised clustering of high-dimensional data using regularized mixture modeling. Brief Bioinform 2021;22. 10.1093/bib/bbaa291. PMC8294591 34293851

[71] Li R , Guan J, Wang Z. et al. A new and effective two-step clustering approach for single cell RNA sequencing data. BMC Genom 2022;23:864. 10.1186/s12864-023-09577-x. PMC10636845 37946133

[72] He-Ming C , Xiang-Zhen K, Jin-Xing L. et al. Joint CC and Bimax: a biclustering method for single-cell RNA-seq data analysis. Bioinformatics Research and Applications. ISBRA, vol. 13064, pp. 499–510. Springer, Cham: Springer-Verlag, 2021. 10.1007/978-3-030-91415-8_42.

[73] Qi R , Ma A, Ma Q. et al. Clustering and classification methods for single-cell RNA-sequencing data. Brief Bioinform 2020;21:1196–208. 10.1093/bib/bbz062. 31271412 PMC7444317

[74] Ntranos V , Kamath GM, Zhang JM. et al. Fast and accurate single-cell RNA-seq analysis by clustering of transcript-compatibility counts. Genome Biol 2016;17:112. 10.1186/s13059-016-0970-8. 27230763 PMC4881296

[75] Saelens W , Cannoodt R, Saeys Y. A comprehensive evaluation of module detection methods for gene expression data. Nat Commun 2018;9:1090. 10.1038/s41467-018-03424-4. 29545622 PMC5854612

